# The purification of reduced β2-glycoprotein I showed its native activity in vitro

**DOI:** 10.1186/s12944-017-0555-x

**Published:** 2017-09-13

**Authors:** Saijun Zhou, Ming Lu, Jiantong Zhao, Shuaihui Liu, Xin Li, Rui Zhang, Hongyan Liu, Pei Yu

**Affiliations:** Key Laboratory of Hormones and Development (Ministry of Health), Tianjin Key Laboratory of Metabolic Diseases, Tianjin Metabolic Diseases Hospital and Tianjin Institute of Endocrinology, Tianjin Medical University, Tianjin, 300070 China

**Keywords:** β2-glycoprotein I, Reduced β2-glycoprotein I, Thioredoxin-1, GSH, Diabetic vascular disease, Oxidative stress

## Abstract

**Background:**

New evidence has shown that reduced β2-glycoprotein I (β2GPI) has anti-oxidative stress and anti-inflammatory activity. However, the details are still poorly understood. This study aims to prepare stable reduced β2GPI with its native bioactivity in vitro.

**Methods:**

Human β2GPI was purified from plasma first with perchloric acid precipitation and then purified with a series of chromatography methods including Sephadex G-25 desalting, SP HP, AF-heparin HC-650 M, and Sephacryl S-200. The purified human β2GPI was reduced with thioredoxin-1 (TRX-1) activated by DL-dithiothreitol (DTT). Glutathione (GSH) was selected to block the free thiols in reduced β2GPI. LC/MS was used to verify the location of free thiols. Western blot analysis was used to detect β2GPI immunoreactivity. MTS and flow cytometry were conducted to investigate its biological effect on oxidative-stress-induced death of human umbilical vein endothelial cells (HUVECs). The levels of tumour necrosis factor-alpha (TNF-α),interleukin-6 (IL-6) interleukin-10 (IL-10),interleukin-12P70 (IL-12P70),interferon-gamma (IFN-γ) and monocyte chemoattractant protein −1(MCP-1) in mouse serum were quantified to assess its anti-inflammatory activity in lipopolysaccharide (LPS)-mediated systemic inflammation.

**Results:**

We obtained approximately 10 mg β2GPI (purity 98.7%) from 200 ml plasma. The protein yield was 0.05 mg/ml plasma. β2GPI was then reduced by TRX-1/DTT in vitro; the free thiols were detected on Cys288 and Cys326 in domain V of β2GPI. The GSH blockage stabilized the reduced β2GPI in vitro. This reduced β2GPI can be recognized by the anti-β2GPI antibody, can significantly reduce the death of HUVECs after H_2_O_2_ treatment and can significantly decrease the levels of TNF-α, IL-6,IFN-γ and MCP-1 in mice upon LPS stimulation.

**Conclusion:**

Stable reduced β2GPI can be obtained in vitro by TRX-1 deoxidation followed by the blockage of thiols with GSH. This reduced β2GPI maintains the same immunological activity as oxidized β2GPI and has the ability to counter the oxidative stress induced by H_2_O_2_ in HUVECs and inflammation in LPS-mediated inflammation in mice.

## Background

β2-glycoprotein I (β2GPI), which is also known as apolipoprotein H, is a phospholipid-binding plasma protein that circulates at a concentration of approximately 4 μM. β2GPI is well known as the major antigen of antiphospholipid syndrome and has a dual function as a procoagulant and anticoagulant [1, 2]. Recent studies have shown that β2GPI could also play a role in vascular disease [3–6] as well as the innate immune response [7–9].

β2GPI is composed of five domains and four glycosylation sites connected to its N terminus with a “hook-like” crystal structure [1]. Domains I to IV each have two disulfide bridges, whereas domain V has three disulfide bridges, including a disulfide bridge that incorporates a C-terminal cysteine [1]. Domain V also contains a positively charged lysine-rich region and a hydrophobic flexible loop segment, and both of these regions are required for the binding of β2GPI to negatively charged macromolecules [1]. The disulfide bond in domain V is susceptible to cleavage by the oxidoreductases thioredoxin-1 (TRX-1) and protein disulfide isomerase (PDI), leading to the generation of free thiols at Cys288 and Cys326 [3, 10, 11]. This special form of β2GPI is called reduced β2GPI. Therefore, β2GPI exists in two forms (reduced β2GPI and oxidized β2GPI) in plasma, and the main form of β2GPI in plasma is the free thiol form [10]. In recent years, reduced β2GPI was found to have distinct functions from the oxidized β2GPI [3, 4, 9]. Reduced β2GPI has been shown to play a protective role against oxidative stress-induced vascular endothelial cell death, indicating that it has an anti-oxidative stress function. Its anti-oxidative stress function was further confirmed in age-related macular degeneration in patients [12] and hypoxia-induced retinal angiogenesis in mice [13]. Reduced β2GPI was also found to inhibit macrophage foam cell formation that is induced by oxidized low density lipoprotein (ox-LDL) [4] in vitro and to inhibit vascular lipid deposition and plaque formation by reducing the expression of matrix metalloproteinases/tissue inhibitors of metalloproteinases (MMPs/TIMPs) via the downregulation of the p38 mitogen-activated protein kinase (p38 MAPK) signalling pathway in vivo [14], which suggests that reduced β2GPI may alleviate vascular lipid toxicity through an anti-inflammatory mechanism. This anti-inflammatory activity was recently confirmed by our previous study in LPS-mediated systemic inflammation in mice [9].

However, the anti-inflammatory mechanism of the reduced β2GPI is still poorly understood. Although a large proportion of plasma β2GPI exists in free thiol form, this form is unstable in vitro [3]. Until now, the preparation of stable reduced β2GPI, whether by isolating the β2GPI from the plasma or by recombinant methods, has not been reported. Therefore, it is important to obtain a stable product in vitro to understand this special form of β2GPI.

In this study, we prepared stable reduced β2GPI in vitro by a deoxidation reaction and the thiol blockage method and investigated its effect on the oxidative stress-induced death of human umbilical vein endothelial cells (HUVECs) and its anti-inflammatory activity in LPS-mediated systemic inflammation in mice.

## Methods

### Materials

Human plasma was acquired from the Tianjin Blood Centre. The Sephadex G-25 desalting column, SP HP column, AF-heparin HC-650 M column, and Sephacryl S-200 column were purchased from GE Healthcare (USA). Reduced L-glutathione, dithiothreitol, and N-ethylmaleimide were purchased from Sigma (Saint Louis, MO). Recombinant TRX-1 was purchased from R&D Corporation (Minneapolis, MN). The CellTiter 96® AQueous One Solution Cell Proliferation Assay Kit was purchased from BD Biosciences (Franklin Lakes, NJ). Annexin V-FLUOS staining kit was purchased from Roche (Penzberg, Germany). The other materials were purchased from The Tianjin Ruentex Science and Technology Development Co. Ltd. LPS from *E. coli*, (serotype 0111:B4) was purchased from Sigma (Sigma-Aldrich Inc., St. Louis, MO). Male C57BL/6 J mice (6 to 8 weeks old) were obtained from the Experimental Animal Center of Peking University Health Science (Beijing, China). Mouse IL-6 and TNF-α ELISA kits were purchased from Sangon Biological Engineering Technology and Services Co., LTD (Shanghai, China). Human plasma was purchased from the Tianjin Municipal Blood Center (Tianjin, China). Rabbit anti-human β2GPI monoclonal antibody was purchased from Abcam (London, UK). Goat anti-rabbit polyclonal antibody was purchased from Bioworld Technology, Inc. (St. Louis, MO, USA). This study was approved by the Animal Ethics Committee of the Metabolism Disease Hospital of Tianjin Medical University.

### Human plasma precipitation and sample desalting

Human plasma was mixed with perchloric acid (70% volume fraction) at a volume ratio of 1:40, stirred for 30 min at 4 °C and then centrifuged at 1000 rpm at 4 °C to recover the supernatant. The supernatant was reserved, and the pH was titrated to 8.0 with a saturated Na_2_CO_3_ solution.

The cleared supernatant (170 ml) was loaded onto a desalting buffer (50 mM acetate pH 5.2)-preequilibrated G-25 desalting column (XK50 × 20 with 17 cm column height, GE Healthcare, USA) at a flow rate of 20 ml/min. A total of 180 ml of the sample was collected in this step, and the sample was then purified with an SP HP column.

Briefly, the desalted sample was loaded onto a preequilibrated HiTrap SP HP (GE Healthcare, USA) at a flow rate of 3 ml/min in buffer A (50 mM acetate pH 5.2). The column was then washed with buffer A for 2 column volumes (CV), 2% buffer B (50 mM acetate pH 5.2, 1 M NaCl) for 5 CV and eluted with a gradient of 5%–50% buffer B for 20 CV, and 50%–100% buffer B for 5 CV, and then the elution gradient was switched to 100% buffer B for 5 CV. The β2GPI standard sample and protein fractions of every tube were used for SDS-PAGE. According to SDS-PAGE, the protein fractions with a band showed the same profiles as the standard sample. Some samples of mixed proteins were digested with trypsin PNGase F and analysed by LC/MS to verify β2GPI-positive fractions. LC/MS analysis was conducted as previously described [13, 14]. Fractions containing β2GPI were pooled together for AF-heparin HC-650 M column purification.

### AF-heparin HC-650 M column purification

An AF-heparin HC-650 M column (Tricorn 10 × 10 with 9 cm column height, USA; the medium-sized column was purchased from TOSHH) was used for the middle purification step. After the column was equilibrated with equilibration buffer (20 mM Tris 8.0), the pooled samples from the SP HP step were loaded onto the AF-heparin HC-650 M column and washed with buffer A for 2 column volumes (CV), 20% buffer B (50 mM acetate pH 5.2, 1 M NaCl) for 5 CV, 30% buffer B (50 mM acetate 1 M NaCl pH 5.2) for 5 CV, eluted with a gradient of 30%–80% buffer B for 20 CV, and then the elution gradient was switched to 100% buffer B for 5 CV. The fractions were then collected and analysed by SDS-PAGE. The fractions containing the same profile as the standard sample were pooled together.

### Protein clean-up with the S-200 column

Fractions containing the target protein from the last step were concentrated by an Amicon Ultra-15 ml concentrator (10 kDa Centrifugal Filter Unit, Merck-Millipore) to 5 ml and then loaded onto a Superdex-200 column (HiLoad 16/60 Superdex 200 prep grade, GE) that was preequilibrated with equilibration buffer (20 mM PB, 0.15 M NaCl, 10% glycerol pH 7.2). The fractions collected in this step were also analysed by SDS-PAGE. N-glycosylation was verified by the PNGase F enzyme digestion method [15].

### Preparation of reduced β2GPI and blockage method development

- The following solutions were mixed together: 420 μl HBS buffer solution, 40 μl DTT solution (20 mM HBS, 1 mM DTT), and 26 μl TRX-1 solution (0.78 mg/ml), and then incubated at 3 °C for 45 min.
- Approximately 45 μl β2GPI solution (2 mg/ml) was added to 405 μl HBS buffer solution, and the mixed solution was shaken by brief centrifugation.
- β2GPI-HBS mixed solution was then added to the DTT-TRX-1-HBS solution, and the reaction solution was incubated at 37 °C for 1 h.
- Thereafter, the reaction solution was divided into three aliquots, and iodoacetamide solution (40 mM), GSH solution (40 mM), or HBS buffer solution was separately added to each aliquot. The solution was placed at 4 °C to end the reduction reaction, and each reaction was analysed by nonreducing SDS-PAGE.

To block the free thiol form of β2GPI, cysteamine or GSH was added to the 50 μl reaction with final concentrations of 40 and 80 mM, respectively. The reaction was then incubated at 37 °C for either 2 h or overnight. Aliquots (2 μg) of samples from each step and time point were analysed by nonreducing SDS-PAGE.

### LC/MS analysis of the free thiol form of β2GPI

To develop an LC/MS-based method to analyse the reduced form of β2GPI, the purified β2GPI was pretreated using two different conditions: A) 2 μg protein was denatured in 50 μl denaturing buffer (10 mM DTT, 0.1% RapiGest (Waters Corp.), 50 mM NH_4_HCO_3_) for 30 min at 60 °C; B) 2 μg protein was denatured as in (A), and iodoacetamide (IAA) was then added to a final concentration of 20 mM and incubated at RT for 30 min to fully alkylate the reduced cysteine. The samples of the two conditions were enzymatically digested for 4 h at 37 °C with trypsin at a 20:1 (*w*/w, protein:enzyme). Trifluoroacetic acid (TFA) was added to each sample to the final of 0.5% to stop the enzyme digestion. The clarified samples were used for LC/MS analysis. LC/MS analysis was conducted as previously reported [16, 17].

The samples from the TRX reduction and alkylation blockage reactions were digested in 0.1% RapiGest, 50 mM NH_4_HCO_3_, and the samples from the TRX reduction and GSH reactions were digested with trypsin for 4 h at 37 °C at a 20:1 (*w*/w, protein:enzyme). Trifluoroacetic acid (TFA) was added to each sample to a final concentration of 0.5% to stop the enzyme digestion. The clarified samples were used for LC/MS analysis.

### Western blot analysis for the immunoreactivity of reduced β2GPI

The aliquots (5 ng per lane) of the total protein were resolved on a Nu-PAGE TM 4–12% Bis-Tris gel and blotted onto a nitrocellulose membrane. The membrane was blocked with 2% BSA in TBST (20 mM Tris-HCl pH 7.6, 137 mM NaCl, and 0.01% Tween-20) for 1 h at RT, followed by incubation with anti-β2GPI antibody overnight. After washing with TBST, the membrane was re-probed with HRP-anti-rabbit IgG (1:1000) in 2% BSA in TBST for 2 h at RT. After exposure in the darkroom, peroxidase activity on PVDF membranes was visualized on X-ray film with an ultraviolet transmission analyser.

### Effect of reduced β2GPI on oxidative stress-induced cell death in human endothelial cells

HUVECs were obtained from freshly delivered umbilical cords after informed consent. Cells were cultured with EGM or M199 plus 20% FBS ECGP and incubated at 37 °C in 5% CO_2_. Endothelial cells were identified by their typical cobble-stone morphology and by immunostaining with anti-CD31 antibody, as previously described [6]. Experiments were conducted using HUVECs between passages 3 and 4. HUVECs were seeded at a density of 1 × 10^5^ cells/mL in 96-well or 24-well plates and grown to confluency over 24–48 h. Cells were grown to confluency and treated with 10 nM H_2_O_2_ as previously described [4]. After overnight incubation in M199/0.1% FCS, the cells were washed twice in M199/0.05% BSA; then, 1 μM of β2GPI or reduced β2GPI was added to the medium and cells were incubated at 37 °C for 30 min. The cells were then transferred to a 1.5 ml Eppendorf tube containing H_2_O_2_ diluted in HBS to a final concentration of 10 mM and incubated at 37 °C for 30 min. H_2_O_2_ was then deactivated with catalase, cells were incubated overnight at 37 °C with M199/FCS (10%), and cell viability was quantified with MTS as previously described [6]. To investigate the effect of reduced β2GPI on cell proliferation, we used trypan blue dye to count the number of living cells. The cells were transferred to 24-well plates and overgrew the plates. After the cells were collected, 0.4% (*w*/*v*) trypan blue dye was added, and using a haemocytometer under the optical microscope, the number of living cells (3 wells) was counted. Cell apoptosis was assessed using flow cytometry. Cells were digested with trypsin and collected by centrifugation (2000 rpm, 5 min). Binding buffer (500 μl) was added to cells. Then, 5 μl annexin V-FITC and 5 μl propidium iodide were added to the binding buffer. Samples were incubated in the dark for 10 min at RT, and then flow cytometric analysis was performed. The excitation wavelength was 488 nm. The emission wavelength was 530 nm.

### Effect of reduced β2GPI on LPS-mediated systemic inflammation in mice

Sixteen male mice in each group were housed in a specific pathogen-free environment, and their health status was regularly monitored. Purified plasma β2GPI (2 μM) or reduced β2GPI with 1 nM LPS were preincubated for 15 min at 37 °C before injection into mice [7]. Then, male mice were injected with 1 μg/g body weight (gbw) with LPS from *E. coli* (Sigma-Aldrich) in GSH (80 mM) or the same volume of sterile saline through tail vein. Six hours post-injection, mice were euthanized, and approximately 1 ml of blood was collected by cardiac puncture. Serum was collected by centrifugation of clotted blood at 1500 g for 10 min to be used in a cytokine assay. The supernatants were separated, and the levels of IL-6, IL-10, IL-12p70, IFN-γ, TNF-α, and MCP-1 were determined using a cytometric bead array kit for mouse inflammatory cytokines (BD Biosciences) according to the manufacturer’s instructions. Briefly, beads with distinct fluorescence intensities are coated with antibodies that specifically react with each of the cytokines and are detected by phycoerythrin-conjugated antibodies, which produces a fluorescent signal in proportion to the amount of bound analyte. The bead populations were mixed together to form the bead array, which was resolved in a FACSAria TM flow cytometer, and the beads were differentiated by their distinct spectral characteristics. Standard curves were determined for each cytokine from 20 to 50,000 pg/ml. The data were acquired on a FACS Aria TM flow cytometer (BD Biosciences) and analysed with FCAP Array software (Soft Flow Hungary, Kedves, Hungary) by applying the 4-parameter curve fit option.

### Statistical analysis

SPSS 11.5 statistical software was used for data processing and analysis. The values for all measurements are expressed as the mean ± standard error of the mean. One-way ANOVA was used to evaluate significant differences. All *P* values were two-sided and were considered statistically significant if they were less than 0.05 (*P* < 0.05).

## Results

### Production of β2GPI

**Fig. 1:**
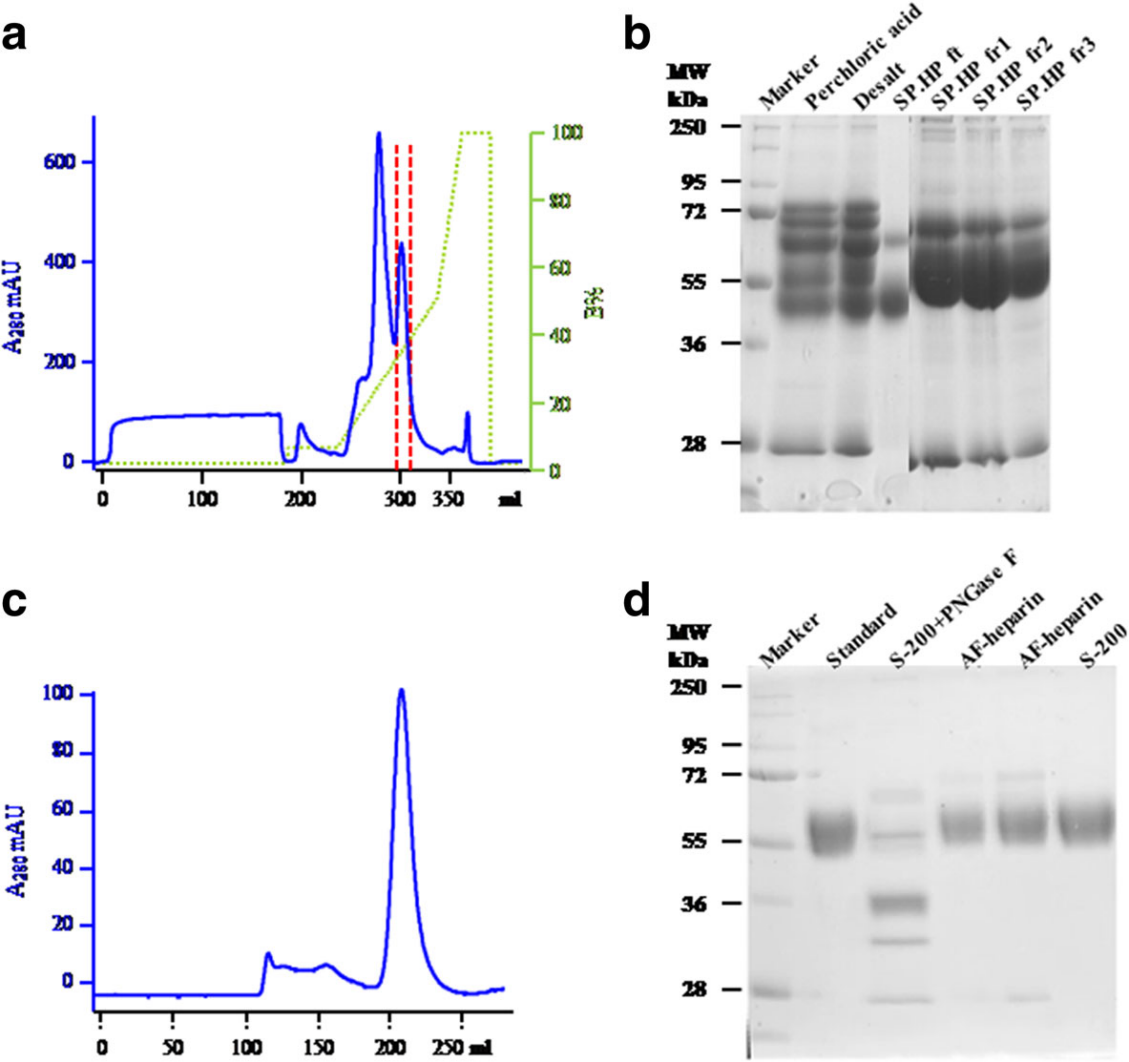
Purification of β2GPI. Human plasma was treated with perchloric acid, desalted with G-25 column, and purified with a series of chromatography steps on an SP HP column, AF-heparin HC-650 M column, and S-200 column in sequence. After purification with the SP HP column (**a**), the sample fractions (**b**) were analysed by SDS-PAGE. Marker: protein standard marker (Bio-Rad cat. 161–0374), perchloric acid: supernatant after perchloric acid precipitation, Desalt: G-25 desalted sample, SP HP ft.: the flow-through of SP HP, SP HP fr1–3: the pooled elution fractions of SP HP. After further purification with the AF-heparin HC-650 M column and S-200 column (**c**), the sample fractions were analysed by SDS-PAGE (**d**). Marker: protein standard marker (Bio-Rad cat. 161–0374), PNGase F: SP digestion, AF-heparin: fractions of AF-heparin chromatography step, S-200: pooled fractions of S-200 step

In the SP HP chromatography step, β2GPI was eluted as the second main peak (Fig. 1a). The purity of the pooled peak showed a dramatic increase in purity but still contained several impurities (Fig. 1b). After AF-heparin HC-650 M and gel filtration purification, the sample showed a homogenous peak on the S-200 column (Fig. 1c), and the purity was as good as that of the commercial standard (Fig. 1d). Consistent with reports of β2GPI containing several N-glycosylation modifications, the purified protein that was obtained after PNGase F digestion showed smaller bands by SDS-PAGE (lane 3, Fig. 1d). Finally, 10 mg β2GPI was purified from 200 ml human plasma. The purity of β2GPI was approximately 98.7%. The protein yield was 0.05 mg/ml plasma. The purified protein was digested by trypsin and followed by LC/MS-based peptide mapping to confirm the sequence.

### Free thiols in reduced β2GPI are induced and blocked by GSH

**Fig. 2:**
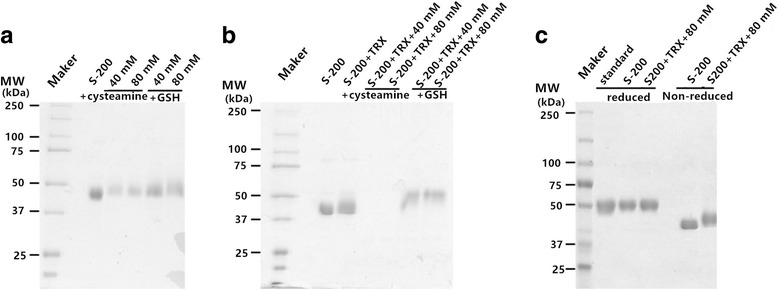
The method development for reduced from β2GPI production and its comparison with native β2GPI. **a** Purified β2GPI could not reduced by cysteamine or GSH alone. **b** Purified β2GPI could reduced by thioredoxin-1 (TRX-1). **c** Reduced β2GPI blocked by GSH under reduced and non-reduced SDS-PAGE conditions. Purified β2GPI of the S-200 step (lane 2) was reduced by thioredoxin-1 (TRX-1) and then cysteine with cysteamine (lanes 3 and 4 with final concentrations of 40 and 80 mM, respectively, at 37 °C for 2 h) and GSH (lanes 5 and 6 with final concentrations of 40 and 80 mM, respectively, at 37 °C for 2 h) blockage (**a**). Purified β2GPI of S-200 step (lane 2) was reduced by thioredoxin-1 (TRX-1) and then cysteine with cysteamine (lanes 3 and 4 with final concentration of 40 and 80 mM, respectively, at 37 °C overnight) and GSH (lanes 5 and 6 with final concentration of 40 and 80 mM, respectively, at 37 °C for 2 h) blockage (**b**). The final comparison of commercial standard β2GPI (lane 2) with purified native (lane 3) and GSH blocked (lane 4) under reduced and non-reduced SDS-PAGE conditions. 5 μg protein were loaded into each lane

In the free cysteine blockage step, two different blockage reagents, cysteamine and GSH, were compared at two different time points (2 h and overnight at 37 °C). After blockage for 2 h, the MW was slightly different from the native form, but the difference was not obvious (Fig. 2a). After blockage overnight, the target protein disappeared under the cysteamine-blockage condition. In addition, the GSH-blockage condition resulted in an obvious MW increase (Fig. 2b). The reduced and GSH-blocked forms were compared and analysed by both reducing and nonreducing SDS-PAGE. When analysed by reducing SDS-PAGE, the native and reduced forms had the same MW; however, when analysed by nonreducing SDS-PAGE, the reduced and blocked forms had a higher MW (Fig. 2c).

**Fig. 3:**
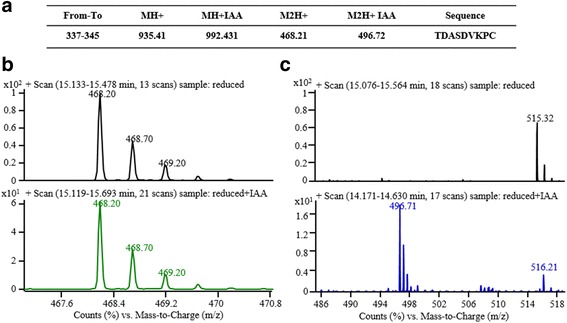
LC/MS-based peptide characterization method development for reduced β2GPI. The theoretical MWs of the target peptide (TDASDVKPC) in different charge and alkylation form was described (**a**). The digestion product after reduction and reduction plus alkylation were compared (**b** & **c**). b shows the reduced peptide form of MH^2+^, while c shows the alkylated peptide form of MH^2+^. Especially in Fig. c, alkylated peptide can be characterized only in the reduction plus alkylation sample, which implies that if the cysteine is blocked with GSH, then it will not be alkylated

Based on the protein sequence and the trypsin theoretical digestion, the information for the target peptide (**TDASDVKPC**) is described in Fig. 3a. The protein sample after reduction contained only the reduced peptide (Fig. 3b & c, upper); however, the protein sample after reduction and alkylation exhibited an alkylated peptide but a decreased reduced peptide (Fig. 3b & c, lower). In this way, the method can be used in reduced and GSH-blocked protein analysis.

**Fig. 4:**
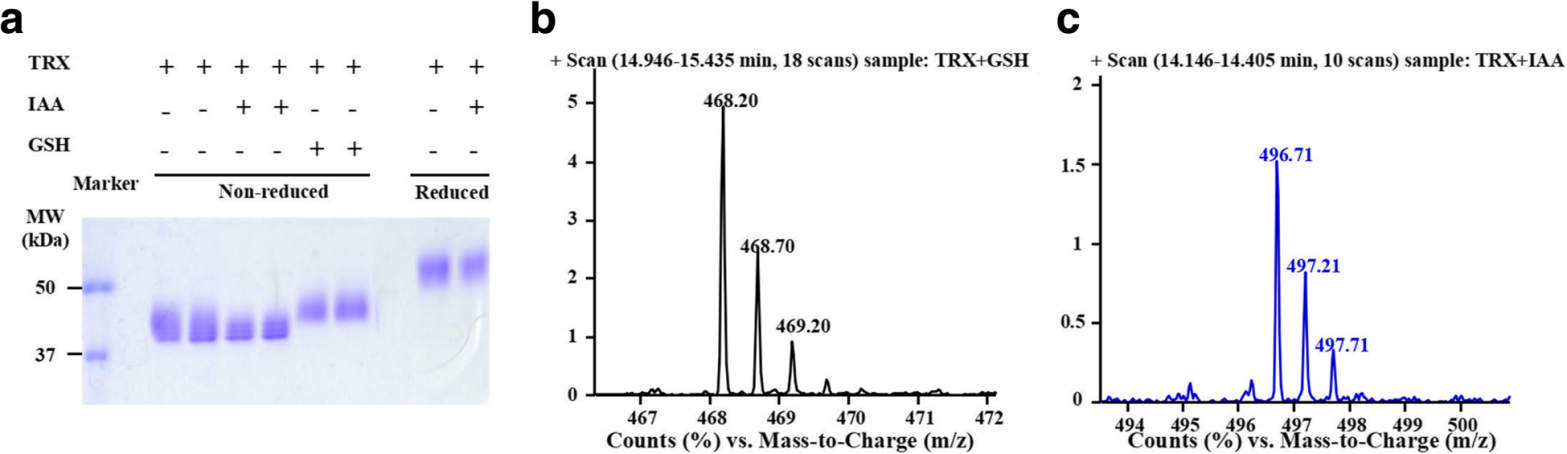
The final LC/MS characterization of the reduced and GSH-blocked β2GPI. **a** Reduced β2GPI blocked by GSH showed different MWs non-reduced SDS-PAGE conditions. **b** The LC/MS ion peak of GSH blocked reduced β2GPI. **c** The LC/MS ion peak of IAA blocked β2GPI. The sample conditions and the profile comparison on SDS-PAGE was visualized (**a**). The samples in lane 7 and lane 5 were digested and analysed by LC/MS. The reduced and GSH-blocked β2GPI sample shows only the reduced peptide form of MH^2+^ (**b**), while the reduced and alkylated β2GPI sample shows only the alkylated peptide form of MH^2+^. These results proved that our reduced and GSH-blocked β2GPI was produced in the right form

The samples from different conditions of the reduced and GSH-blocked protein analysis step are shown in Fig. 4a. Based on the LC/MS analysis, the reduced and GSH-blocked protein resulted in only a reduced peptide, but the reduced and alkylated protein resulted in only an alkylated peptide. The M^2H+^ = 468.21 ion chromatography and the peak of the reduced and GSH-blocked protein indicated that Cys326 was reduced by TRX. The M^2H+^ = 496.72 ion peak for the reduced and alkylated protein proved that the reduced and alkylated Cys326 peptide exist.

### Immunoreactivity of reduced β2GPI

**Fig. 5:**
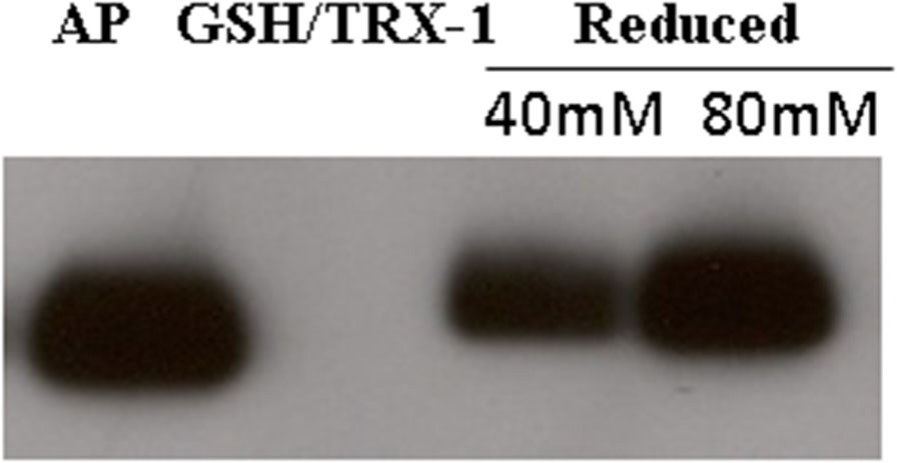
Reduced β2GPI maintained the same immunological activity as oxidized β2GPI. Reduced β2GPI was blocked with GSH (40 mM and 80 mM). AP: purified β2GPI

To further confirm that the reduced β2GPI has maintained its disulfide bridges without disrupting the other domains, an anti-human β2GPI antibody was used for Western blot analysis. The results showed that both oxidized β2GPI and reduced β2GPI can bind mouse anti-human β2GPI antibody (Fig. 5), thus indicating that the reduced β2GPI can still maintain the immunoreactivity of β2GPI and, at least, maintains the original structure of domain I.

### Reduced β2GPI protects HUVECs from oxidative stress-induced cell death

**Fig. 6:**
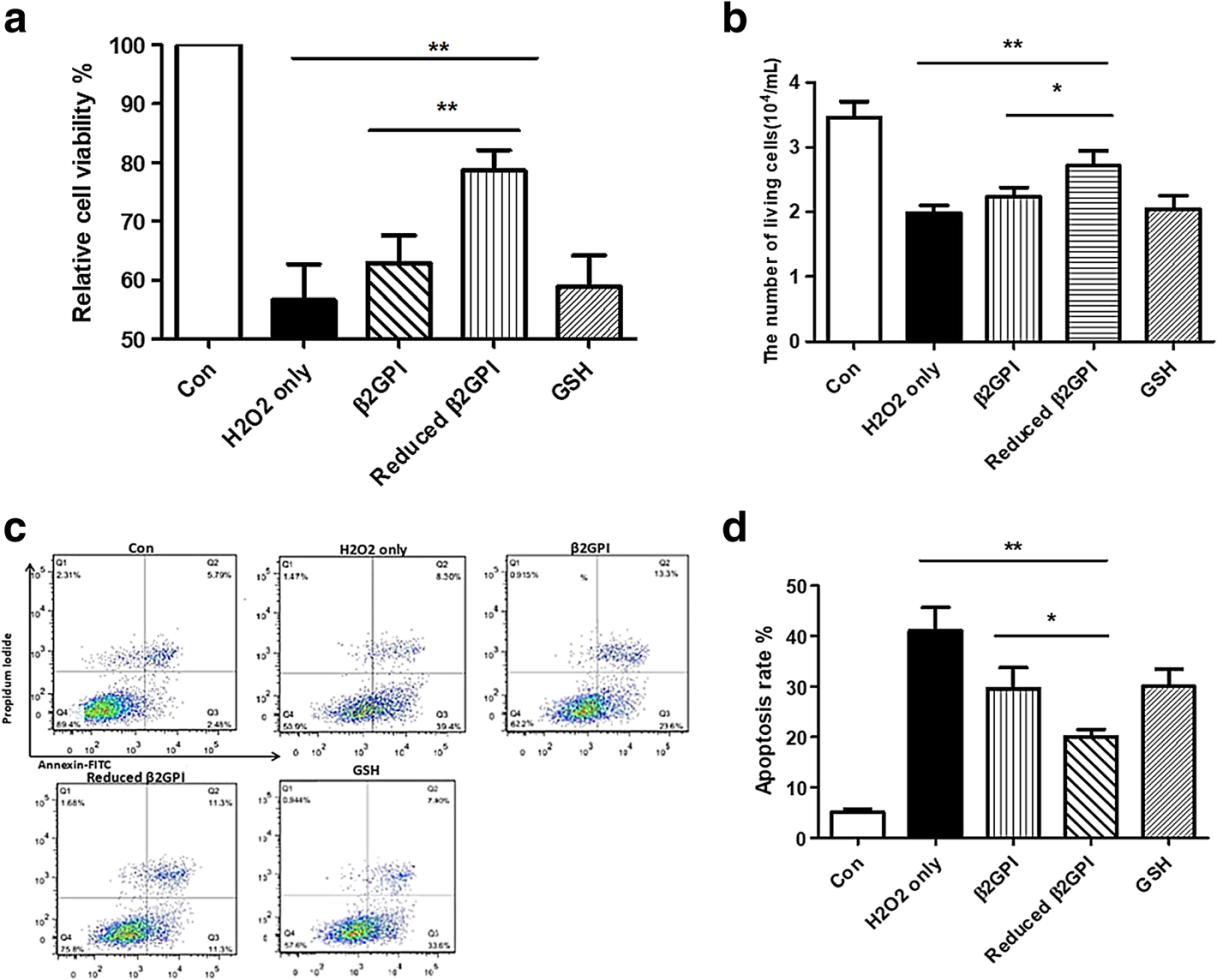
Reduced β2GPI protected HUVECs from oxidative stress-induced cell death. Reduced β2GPI increased the HUVECs cell viability (**a**) and cell number (**b**) upon the H_2_O_2_ treatment. (**c**) The image of the cell apoptosis by FACS. (**d**) Reduced β2GPI protected HUVECs from apoptosis under H_2_O_2_ treatment. HUVECs incubated with β2GPI (1 μM) were pretreated with TRX-1 (1.75 μM) + DTT (35 μM) + GSH (80 mM) for 30 min at 37 °C and then incubated with 4 mM H_2_O_2_ for 40 min at 37 °C. No difference was observed (*n* = 5) between H_2_O_2_-only-treated cells and β2GPI only or TRX-1 (1.75 μM) + DTT (35 μM) + GSH (80 mM). For all panels, *P < 0.05 and **P < 0.001

To confirm whether reduced β2GPI can maintain its biological activity after GSH blockage, we conducted an experiment as previously reported [4]. Our results showed that reduced β2GPI can significantly improve the activity as well as the number of living cells (Fig. 6a and b) and reduce apoptosis (Fig. 6c and d) in HUVECs after H_2_O_2_ treatment.

### Reduced β2GPI has anti-inflammatory activity in LPS-mediated systemic inflammation

**Fig. 7:**
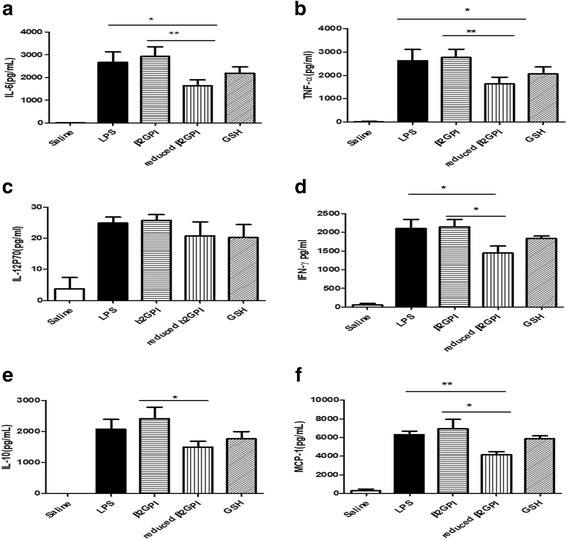
Reduced β2GPI inhibited LPS-mediated inflammation in mice. The effect of reduced β2GPI on the levels of IL-6 (**a**), TNF-α (**b**), IL-12P70 (**c**), IFN-γ (**d**), IL-10 (**e**) and MCP-1 (**f**) in mice serum upon LPS stimulation. Purified plasma β2GPI (2 μM) or reduced β2GPI with 1 nM LPS were preincubated for 15 min at 37 °C before injection into mice at 1 μg/g body weight (gbw) with *E. coli* LPS (Sigma-Aldrich) in sterile saline or the same volume of saline with TRX-1 (1.75 μM) + DTT (35 μM) + GSH (80 mM) through the tail vein. At 6 h post injection, mice were euthanized, and approximately 1 ml of blood was collected by cardiac puncture. Serum was collected and the levels of cytokines (IL-6, TNF-α, MCP-1, IFN-γ, IL-10, IL-12P70) were quantified. Reduced β2GPI significantly reduced the levels of IL-6, TNF-α, MCP-1, and IFN-γ. No difference was observed (*n* = 5) between β2GPI only or GSH and LPS. For all panels, *P < 0.05 and **P < 0.001

New evidence has revealed that β2GPI plays an important role in the innate immune response [7–9], and reduced β2GPI can relieve LPS-stimulated inflammation in mice [9]. To assess the anti-inflammatory activity of the reduced β2GPI after GSH blockage, an LPS-mediated inflammation experiment was performed as reported in our previous study [9]. The results showed that only reduced β2GPI could significantly decrease the levels of IL-6, TNF-α, MCP-1 and IFN-γin mice serum 6 h after LPS injection (Fig. 7).

## Discussion

Reduced β2GPI was first reported by Professor Krilis’ team in recent years and was found to be the main form of β2GPI in human plasma [3, 11]. Increasing evidence indicates that this free thiol β2GPI plays a protective role in vascular diseases [3–5] and could also function as a scavenger protein for LPS to attenuate inflammation [7, 9]. These new functions of β2GPI were attributed to the anti-oxidative stress and anti-inflammatory activities of the reduced form of β2GPI. However, the mechanism of action of this special form of β2GPI is poorly understood. Until now, the successful extraction of reduced β2GPI from plasma or serum, which impeded the further study of this special form of β2GPI, had not been accomplished. Therefore, obtaining a stable free thiol β2GPI in vitro is a promising solution.

β2GPI is known to be an abundant plasma protein with a molecular weight of approximately 50 kDa, and its concentration in human plasma is approximately 200 μg/mL [2, 18]. Therefore, the isolation of β2GPI from plasma is more economical and more efficient than recombinant protein purification. This study established a simple and effective method to purify β2GPI from human plasma by treating plasma with perchloric acid, followed by a series of chromatography steps, namely, desalting with a G-25 column and purification with an SP HP column, AF-heparin HC-650 M column, and S-200 column. This purification procedure increased the yield of β2GPI from human plasma. The final yield of β2GPI was approximately 10 mg from 200 ml plasma. This procedure also yielded a high purity of 98.7%. Thus, this method could effectively isolate β2GPI with high purity from human plasma.

To prepare the reduced β2GPI, the DTT-activated oxidoreductase TRX-1 was used to cleave the disulfide bridge in β2GPI, as previously reported [3]. A large proportion of plasma β2GPI exists in free thiol form because thioredoxin (TRX) is expressed widely in prokaryotic cells and eukaryotic cells. β2GPI is one component of the TRX system, and the other two components are the TRX reductase (TrxR) and reduced nicotinamide adenine dinucleotide phosphate [3]. This system plays an important role in maintaining the stable redox state in vivo. In mammals, three isozymes of TRX exist: TRX-1 (located in the cytoplasm), TRX-2 (located in the mitochondria), and TRX-3 (located specifically in sperm, Sptrx-3) [19, 20]. The thiol exchange reaction was also reported to occur in β2GPI, with the help of some reductase systems in platelets [21] or vascular endothelial cells [3]. Therefore, using the TRX system to reduce β2GPI has physiological implications. As such, in the present study, we selected TRX-1 as the reducing agent to simulate the physiological transformation of oxidized β2GPI to reduced β2GPI, as previously reported [3, 11]. LC/MS analysis showed that none of the disulfide bonds in domain I-IV of β2GPI was disrupted and that only the disulfide bond formed by Cys288 and Cys326 in domain V was disrupted, along with the formation of corresponding thiols at the points of Cys288 and Cys326. This result corresponded to the molecular bioinformatics analysis of the β2GPI molecular structure that was reported in a previous study [18].

According to the results of molecular bioinformatics analysis, among the 11 disulfide bonds in the molecule, the Cys288-Cys326 disulfide bond in domain V revealed a −/+ right-handed hook structure, and TRX-1 specifically interacts with the disulfide bond within this structure [15, 16]. Furthermore, Cys326 is exposed on the surface of this molecule; thus, this disulfide bond easily interacts with TRX-1. Therefore, the TRX-1 reduction method in vitro is an effective way to produce reduced β2GPI. Other disulfide-reducing agents, such as β-mercaptoethanol, generally reduces most cysteine residues in a non-selective manner. Our results revealed that the β2GPI sample that reacted with DTT or β-mercaptoethanol had a slower electrophoresis rate than the sample that reacted with TRX-1. Western blot analysis failed to detect its immunological activity (data not shown). Western blot analysis only detected β2GPI activity in the activated TRX-1-treated samples, which also indicates that the free thiols in DI-DIV of β2GPI were not disrupted. Thus, neither DTT nor β-mercaptoethanol is able to simulate the physiological thiol conversion reaction in β2GPI. Meanwhile, TRX-1 is not only a physiological reducing reagent but also an effective reducing agent to produce reduced β2GPI in vitro.

LC/MS analysis did not detect any free thiols inβ2GPI before treatment with TRX-1/DTT in the present study, thus suggesting that the β2GPI extracted from plasma existed in the oxidized form. One important reason is that reduced β2GPI cannot maintain its stability in vitro or under oxidative conditions. Figure 3 shows that reduced β2GPI, without thiol blockage, was degraded at 37 °C overnight and was unstable.

In this study, we acquired stable reduced β2GPI in vitro in the following steps: extracting purified β2GPI from plasma, performing the reduction reaction in vitro, and protecting the free thiols of reduced β2GPI using GSH. Figure 3 shows that GSH blockage can stabilize reduced β2GPI compared with cysteamine. GSH is a small physiological molecule that does not cause toxicity and immunogenicity to the human body and could be an ideal thiol blocker for reduced β2GPI.

With the blockage of GSH, reduced β2GPI will maintain its immunoreactivity. Western blot analysis showed that both the reduced β2GPI blocked with GSH and oxidized β2GPI could detect the same antibody. This finding indicated the existence of a disulfide bond in domain I and the structural integrity of domain I in reduced β2GPI because the antibody combined with the specific epitope in domain I of β2GPI.

Reduced β2GPI was identified to have anti-oxidative stress and anti-inflammatory activity [3, 9]. To further verify the biological activity of the reduced β2GPI blocked with GSH, we detected its effect on oxidative stress-(H_2_O_2_)-induced HUVEC cell death and the cytokine levels in LPS-mediated systemic inflammation in mice. The results confirmed that compared with β2GPI or GSH, reduced β2GPI had significantly improved activity, reducing apoptosis and decreasing the IL-6 and TNF-α levels. Therefore, the GSH blocker could not impede the biological activity of reduced β2GPI, and this activity was independent of GSH.

## Conclusion

We prepared stable reduced β2GPI by TRX-1 deoxidation followed by thiol protection with GSH in vitro. This special form of β2GPI with GSH-blocked free thiols maintained the immunoreactivity and bioactivity of reduced β2GPI. This study not only provides an effective way to isolate β2GPI from plasma but also shows a simple method to maintain the stability of reduced β2GPI in vitro, which is important to the further study of the physiological function of reduced β2GPI.

## Abbreviations

β2GPI: β2-glycoprotein I
CV: Column volume
DTT: DL-dithiothreitol
GSH: Glutathione
HUVECs: Human umbilical vein endothelial cells
IAA: Iodoacetamide
IL-6: Interleukin-6
LPS: Lipopolysaccharide
MAPK: Mitogen-activated protein kinase
MMPs: Matrix metalloproteinase
ox-LDL: Oxidized low density lipoprotein
PDI: Protein disulfide isomerase
Sptrx-3: Sperm-specific TRX-3
TFA: Trifluoroacetic acid
TIMPs: Tissue inhibitors of metalloproteinases
TrxR: TRX reductase
